# A Machine Learning Approach to Improve Ranging Accuracy with AoA and RSSI

**DOI:** 10.3390/s22176404

**Published:** 2022-08-25

**Authors:** Tingwei Zhang, Peng Zhang, Paris Kalathas, Guangxin Wang, Huaping Liu

**Affiliations:** 1 School of Electrical Engineering and Computer Science, Oregon State University, Corvallis, OR 97331, USA; 2 School of Software & Microelectronics, Peking University, Beijing 100871, China

**Keywords:** machine learning, ANN, AOA, RSSI, indoor positioning

## Abstract

Ranging accuracy is a critical parameter in time-based indoor positioning systems. Indoor environments often have complex structures, which make centimeter-level-accurate ranging a challenging task. This study proposes a new distance measurement method to decrease the ranging error in multipath environment. Our method uses an artificial neural network that utilizes the received signal strength indicator along with a signal’s angle of arrival to calculate the line-of-sight distance. This combination results in a significant reduction of the error caused by multipath effects that common RSSI-based methods suffer from. It outperforms traditional ranging methods while the implementation complexity is kept low.

## 1. Introduction

The rapid growth of internet-of-things applications has led to an increased demand for indoor positioning systems (IPSs) [1]. It is well known that GPS does not perform well in most indoor environments. Technologies that use ultra-wideband (UWB), radio frequency identification (RFID), Bluetooth low energy (BLE), and WiFi signals have been looked into as part of IPS research [2,3]. IPSs that use UWB signals could achieve centimeter-level accuracy, which is desirable for many applications [4,5,6]. However, the high infrastructure cost and limited effective range of UWB methods make them less competitive than some alternative technologies. RFID positioning systems can be cost-effective but they typically provide coarse positions only. In addition, because of their limited effective range, they require a massive infrastructure to cover the same area, which increases implementation complexity [7].

Compared to UWB and RFID-based IPSs, BLE and WiFi-based IPSs are more cost-effective [8,9]. Initially, these systems suffered from low accuracy, but recent research has bridged the performance gap between them and UWB. BLE and WiFi-based IPSs can be categorized into the following subcategories: Time of Arrival (ToA), Time Difference of Arrival (TDoA), Angle of Arrival (AoA), and Fingerprinting [2,3]. ToA-based IPSs use the time that a signal from a transmitter arrives at a receiver to find the location of the transmitter. ToA requires wide signal bandwidth and strict synchronization between the transmitter and the receiver to achieve a high accuracy [10,11,12]. TDoA-based IPSs measure the difference between multiple ToAs, resulting in a simplified implementation that requires only receiver synchronization. However, the accuracy of this approach still depends on the signal bandwidth [13]. AoA-based IPSs utilize angle information from multiple anchors for localization. These systems do not require synchronization. However, due to the multipath effect, the error of these systems gets higher when the distance between the target and receiver increases [14,15]. A more recent trend is fingerprint-based IPSs. These IPSs utilize fingerprint databases that could be built using data such as channel state information (CSI) and received signal strength indicator (RSSI) [16,17,18], which could be acquired by using commercial devices. The system compares the newly received data with the fingerprints to accomplish the localization task. Given their simplicity, these IPSs have low implementation complexity. However, their accuracy typically lies in range of a few meters.

A ranging system that is capable of achieving centimeter accuracy using commercial WiFi devices was proposed in [19]. However, this system requires a channel hopping mechanism, which makes the system equivalent to UWB-based IPSs and compromises the devices’ ability to transmit data. In this study, we propose a WiFi-based method that is capable of reducing ranging errors under 10 cm without requiring a high signal bandwidth nor a strict synchronization process. The proposed method combines AoA estimation and RSSI ranging with an artificial neural network (ANN) to reduce the multipath effect and make it possible to perform high-accuracy ranging tasks with a single access point (AP).

The rest of this paper is organized as follows. Section 2 summarizes previous works in the area. In Section 3, we explain the system and our methodology. The experimental setup and procedure are presented in Section 4. In Section 5, the result of the experiment and the limitations of the system are discussed. Finally, in Section 6, we conclude this study and discuss the future work of our research.

## 2. Related Work

In this section, we provide some background knowledge for the methods used in this work and summarize previous studies on indoor ranging techniques. This section is split into three subsections: RSSI, AoA, and ANN.

### 2.1. RSSI-Based IPSs

There are two major approaches in RSSI-based IPSs: fingerprinting and ranging. The fingerprinting method uses a pre-stored database to compare the incoming signal and determine the target’s location [20,21,22]. The ranging technique gathers distance information from multiple APs using the path-loss model and then uses the triangulation formula to determine the distance between the transmitter and receiver [23,24]. In [16], Martin et al. introduced an RSSI fingerprint-based IPS. The idea was promising at the time; however, the proposed system lacks accuracy. The median error achieved was 1.5 m. In [17], Achroufene et al. proposed a method that improves the accuracy of RSSI-based ranging using belief function theory, which improved the reliability and accuracy of the RSSI-based ranging to a median error of 1.13 m. In [18], Biehl et al. proposed a fingerprint-based IPS, with the help of a classification algorithm. It managed to maintain a median error within the sub-meter level, specifically 0.94 m.

### 2.2. AoA-Based IPSs

AoA-based IPSs determine the target’s location using a cross-section created from multiple angular information. This angular information is calculated by exploiting the phase difference between elements on an antenna array in various locations. In [25], Chen et al. developed an IPS that utilizes angular signature from AoA estimation as fingerprint, achieving an accuracy with median error in the range of 1.5 m. In [26], Kumar et al. proposed a new method to emulate synthetic aperture radar on hand-held devices to perform indoor positioning tasks, managing to achieve an accuracy with error in the range of 0.3 m. In [27], Xiong et al. introduced a method to suppress the multipath component within the AoA spectrum, thus increasing the accuracy of the system. The proposed work showed an accuracy with median error of 0.23 m; however, it uses an antenna array with sixteen antennas, which significantly increases the system cost.

### 2.3. ANN-Based IPSs

In an indoor environment, there are countless propagation paths due to the multipath effect. These propagation paths can contribute both constructive and destructive effects on the received signal. This makes RSSI not strictly follow its path loss model. For this reason, precise ranging based on signal power becomes a challenging task. To address this issue, ANNs have been introduced in recent studies. Unlike traditional methods that rely on a fixed model, ANNs adjust their weights internally to find a best fit for the majority of samples in the dataset. This makes ANN-based methods more suitable to mitigate multipath effects. In [28], Adege et al. proposed a fingerprinting-based IPS that uses the back-propagation ANN and KNN algorithms. Compared to the conventional fingerprinting IPS, their work shows a significant improvement in accuracy with a median error of 0.5 m. In [29], Ibrahim et al. evaluated the performance of multiple ANN algorithms for improving ranging accuracy using RSSI. The simulation result of this work shows that with the help of ANN, the ranging error is rapidly reduced solely with RSSI information. Adhikari et al. also exploited the ANN with a recursive least-squares technique for reducing the positioning error using RSSI [30]. Their simulation results show an extraordinary improvement in terms of accuracy with median error down to 0.035 m. However, the method uses 10 APs, which is complex and costly.

Table 1 summarizes the methods and the median errors these methods have achieved.

## 3. Methodology

**Figure 1:**
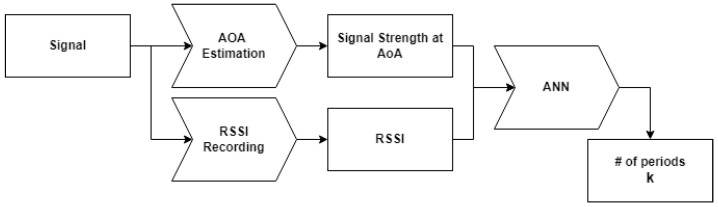
Workflow of the proposed system.

In this work, we propose a new high-precision indoor ranging system that comprises an RSSI recording block, an AoA estimation block, and an ANN block as shown in Figure 1.

Unlike conventional ranging methods that use time measurements to estimate the direct signal’s path length, our method uses the periods that the signal traveled as described by

(1) D=(T+ϕ/2π)×λ

where *T* is the number of periods that the signal has traveled, ϕ is the phase of the received signal and λ is the wavelength of the signal. As Equation (1) shows, with a short wavelength, the systematic error from inaccurate phase information is one wavelength. In this work, we use a signal with a central frequency of 5.52 GHz, which has a wavelength of 0.0543 m.

### 3.1. RSSI

RSSI-based methods convert RSSI to distance information using the path loss model expressed as

(2) $$PL=PL\left(d_{0}\right)+10\gamma \log_{10}\frac{d_{tr}}{d_{0}}+X_{g}$$

where PL is the total path loss in decibels, γ is the path loss exponent, $d_{tr}$ is the distance between the transmitter and the receiver, $d_{0}$ is the reference distance (for example, 1 m or 10 m for indoor environments), $PL(d_{0})$ is the path loss at the reference distance $d_{0}$, γ is the path loss exponent, and $X_{g}$ is a normal random variable with zero mean [31]. Due to the multipath effect, the received signal in an interior setting becomes very noisy. This interference has a great impact on RSSI’s ranging accuracy [17]. In order to obtain accurate distance information using RSSI, first we need to extract the line-of-sight (LoS) signal’s power from the RSSI data. We do this by using the spatial spectrum provided by the AoA estimator.

### 3.2. AoA Estimation

**Figure 2:**
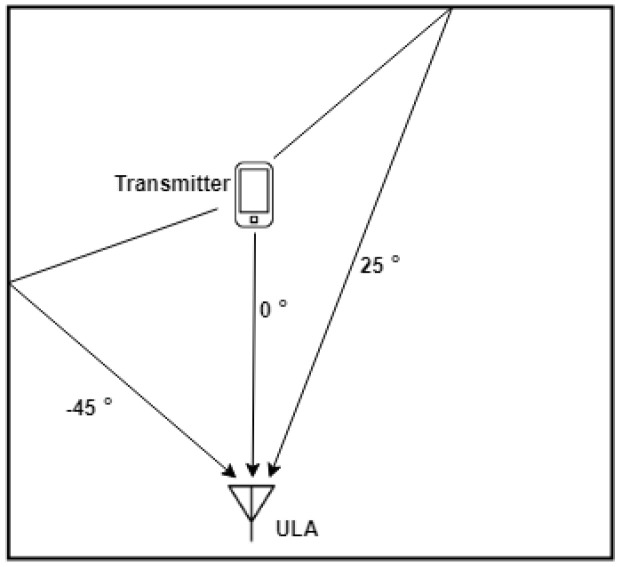
Simulation scenario.

**Figure 3:**
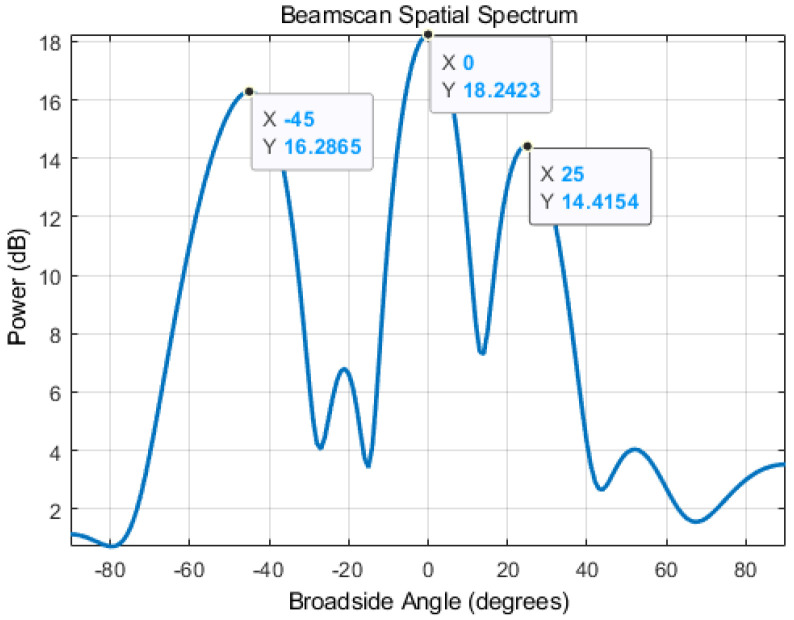
Simulated AoA Estimation Spectrum.

AoA estimation detects the angle that has the highest signal strength in space. In beamforming-based AoA estimation, this is done by evaluating the power of the received signal from all directions [32,33]. The received signal y(t) in the indoor environment can be seen as a weighted sum of multiple signals. Let us assume that there are *m* antennas on an uniform linear array (ULA), and the distance between two adjacent antennas is *d*. For an indoor environment that has a total of *L* propagation paths, the signal model at the *m*th antenna at a given time *t* is given as

(3) $$y\left(t\right)=\sum_{l=0}^{L-1}a\left(\theta_{l}\right)s\left(t\right)$$

where $\theta_{l}$ is the broadside angle of the *l*th propagation path, s(t) is the source signal, and $a(\theta_{l})$ is the steering vector expressed as

(4) $$a\left(\theta_{l}\right)=e^{-j\frac{2\pi }{\lambda }\left(m-1\right)d\sin\theta_{l}}$$

and the model of beamscan is given as

(5) $$P_{BeamScan}\left(\theta \right)=a^{H}\left(\theta \right)R^{-1}a\left(\theta \right)$$

where R is the autocorrelation matrix of the received signal [34]. Using MATLAB, we simulated the conventional beamforming method to verify its ability to describe the spatial energy distribution of a signal. As depicted in the example shown in Figure 2, in an indoor environment, there exist three possible propagation paths, with the direct path signal from $0^{\circ }$ and two reflections from $-45^{\circ }$ and $25^{\circ }$. To simulate this scenario, we set three signal sources in the simulation with distinct signal strengths. The signal strength from $-45^{\circ }$ is set to be 20% lower than the signal from $0^{\circ }$, and signal strength from $25^{\circ }$ to be 40% lower than $0^{\circ }$. As demonstrated in Figure 3, the beamforming-based AoA estimator can distinguish between the signals coming from different angles and their corresponding power level. Our method utilizes this information to assist extracting the correct LoS data from the RSSI.

### 3.3. ANN

Due to the multipath effect, the acquired signals in an indoor environment have complex mathematical models; thus, using an ANN is the most efficient way to process them [35]. In this study, we developed an ANN that takes the RSSI data and the AoA estimation and calculates the number of periods the signal traveled from the transmitter to the receiver. The properties of the proposed ANN are given in Table 2.

**Figure 4:**
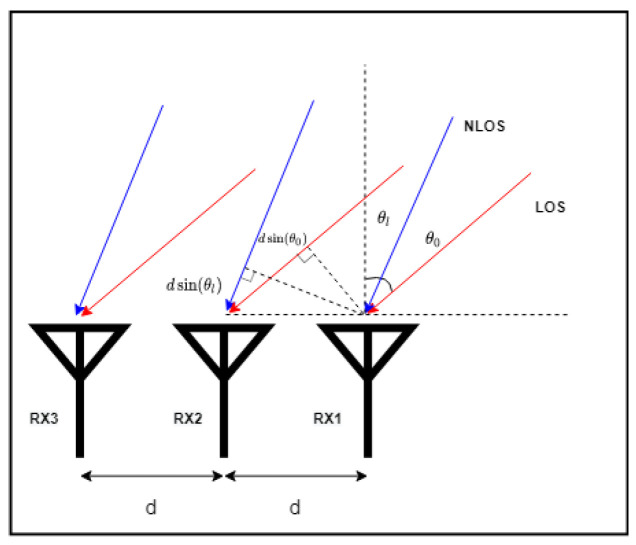
Example of additional RSSI attenuation on antenna array.

Initially, the ANN extracts the correct RSSI data using the AoA estimation spectrum (as shown in Figure 3). Then, the ANN converts the RSSI data to the number of periods the signal traveled. The relationship between RSSI and signal intensity from AoA estimation can be expressed as Equation (6).

(6) $$RSSI=w\times P_{AoA}\left(\theta_{0}\right)+\Phi$$

where *w* is a weight factor, $P_{AoA}$ is the signal power at the LoS angle $\theta_{0}$ taken from the AoA estimation spectrum and Φ is the power change caused on the direct signal by the multipath effect. As shown in Figure 4, for an RSSI recorder with three antennas, the RSSI values received on RX2 and RX3 suffer from additional path loss compared to RX1. Similar to the principle of AoA estimation, the additional loss is caused by the additional distance the signal travels between different antennas.

For a receiver with *m* antennas, the power of the received signal at the *m*th antenna can be expressed as Equation (7)

(7) $$RSSI_{m}=w\times P_{AoA}\left(\theta_{0}\right)+\Phi +\Delta PL\left(m\right)$$

where ΔPL is the additional path loss on the *m*th antenna. These additional RSSI changes on antennas include both LoS and non-line-of-sight (NLoS) scenarios. Changes in RSSI between antennas are caused by different propagation distances from the same signal source. As a result, $\Delta PL_{LoS(m)}$ and $\Delta PL_{NLoS(m)}$ are proportional to the power received from LoS and NLoS paths on RX1, which is given as

(8a) $$\begin{array}{cc} \Delta PL_{LoS}\left(m\right) & =\beta_{1}w\times P_{AoA}\left(\theta_{0}\right) \end{array}$$

(8b) $$\begin{array}{cc} \Delta PL_{NLoS}\left(m\right) & =\beta_{2}\times \Phi . \end{array}$$

Given $P_{AoA}$ and multiple RSSI values, the ANN then adjusts its internal weights to find the best combination of *w*, $\beta_{1}$, $\beta_{2}$, and Φ that works for most samples in the dataset that satisfy (6) and (9) below:

(9) $$\Delta PL=\beta_{1}w\times P_{AoA}\left(\theta_{0}\right)+\beta_{2}\times \Phi$$

**Figure 5:**
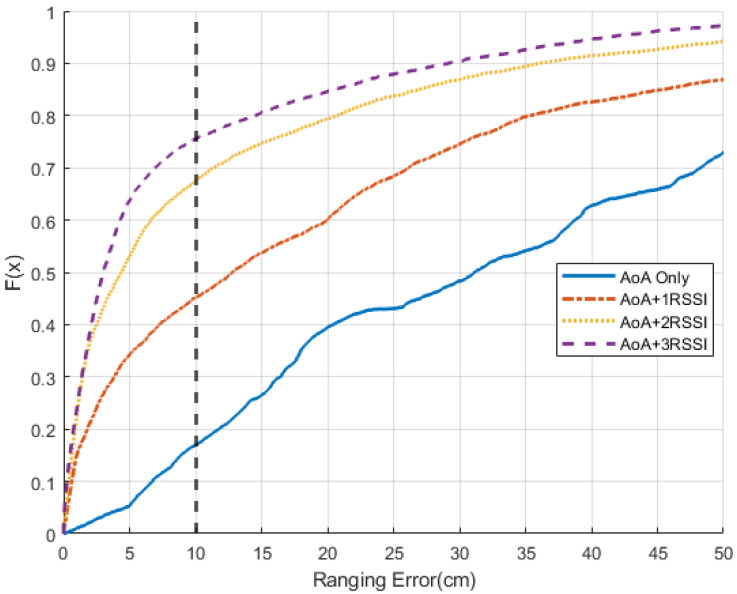
Error comparison between differentinput.

Once *w* is found, the ANN can estimate the power of the LoS signal by multiplying *w* with $P_{AoA}$. Then, the proposed ANN can further convert the power information to distance and transform it into a number of periods *T*. Figure 5 demonstrates how the ranging error is reduced based on the amount of RSSI signals used as input to ANN. With at least two RSSI values, the ranging error can be significantly reduced.

## 4. Experiment

### 4.1. Experimental Devices

Three devices are used in this experiment: two TP-LINK WR2543N wireless routers and an AD-9361 evaluation board. The router with a single antenna serves as the transmitter; another router with three antennas serves as the RSSI recorder. The AD-9361 evaluation board with a four-element antenna array is used for the signal waveform-capturing process. Signals are transmitted through channel 104 with a center frequency of 5.52 GHz and a bandwidth of 20 MHz during the experiment.

### 4.2. Experimental Setup

**Figure 6:**
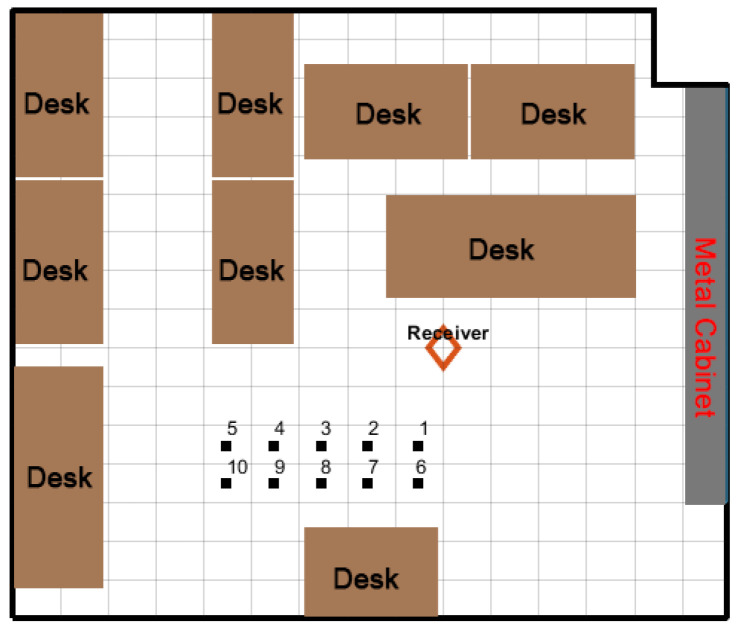
Layout of the test field.

The experiment took place in a lab environment with dimensions (L×W) of 9.6 m × 9.1 m. The layout of the lab environment is shown in Figure 6. Ten locations were randomly selected with a maximum LoS distance of 3.78 m. The exact coordinates of selected locations are included in the Appendix A.

### 4.3. Experimental Process

The experimental process has following steps:

1. Create an indoor environment and record the geometry of the selected transmitter location points, i.e., their coordinates, distance, and theoretical AoA.
2. Record the RSSI and the signal waveform.
3. Translate the signal waveform to the AoA spectrum and construct a dataset for the training stage.
4. Use the dataset to train the ANN and to obtain the weight factors of the model.
5. Estimate the number of periods *T* using the ANN and signal parameters.

The ANN’s performance depends on the size of the dataset. To ensure the accuracy of the network, we collect 500 samples at each selected location. Thus, the dataset has a total of 5000 samples. Each sample consists of four inputs, AoA strength, RSSI1, RSSI2, and RSSI3 and one output, the number of periods the signal traveled. We build the ANN model in Matlab. The Bayesian Regularization algorithm with a minimal gradient of $10^{-7}$ is used to train the network [36]. We divide the dataset randomly into two subsets, each having 2500 samples. We use one dataset to train the network. After the training stage, the ANN was tested against the second set of data to see if it can give the expected results. With 30 layers, the ANN achieves optimal performance resulting to a median error of 0.037 m.

## 5. Result and Limitations

**Figure 7:**
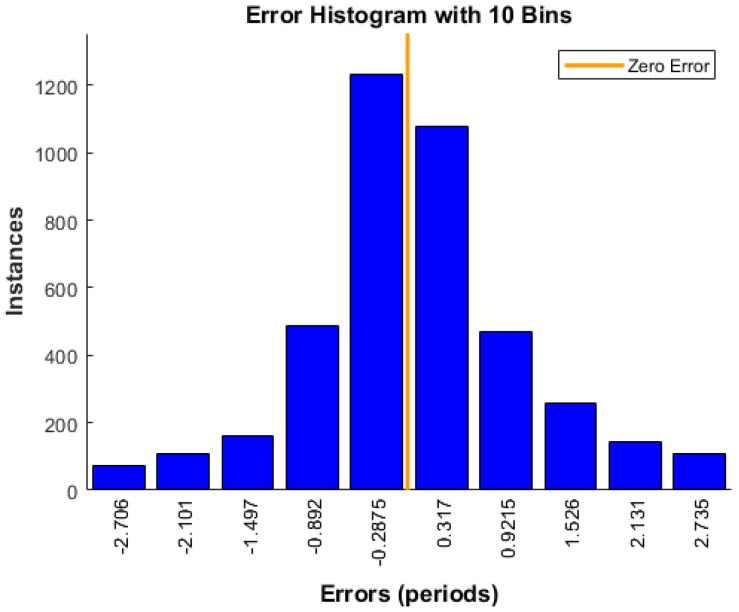
Error Histogram.

**Figure 8:**
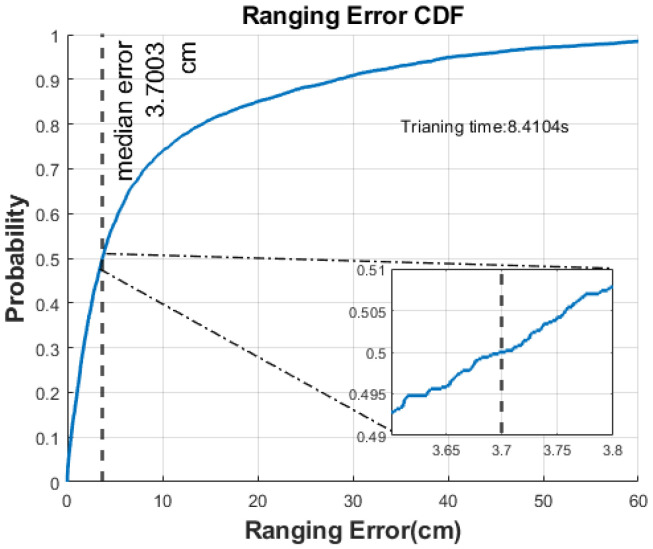
Ranging error CDF.

The performance of the ANN is evaluated based on its error, which is calculated by Equation (10)

(10) $$\varepsilon_{T}=T-T^{\prime }$$

where *T* is the actual number of periods, and $T^{\prime }$ is the number of periods predicted by the ANN. The mean squared error (MSE) in predicting the number of periods by the ANN is 8.99, with a standard deviation of 17.5. With a total of 5000 samples, the error histogram of the ANN provided in Figure 7 shows that more than 60% of the predictions have an error less than 0.5 periods. The ranging error can then be calculated using Equation (11).

(11) $$\varepsilon_{D}=\left(T-T^{\prime }\right)\times \lambda .$$

where λ is the wavelength of the system (5.43 cm in this work). The cumulative distribution function (CDF) in Figure 8 shows that 75% of the errors were less than 0.1 m with a median error of 0.037 m and a mean error of 0.092 m.

However, the current system is far from perfect. To begin with, the suggested system is still in its prototype stage; the RSSI recorder and signal waveform receiver are not synced. This increases the failure rate of the signal acquisition procedure, as two devices cannot begin recording simultaneously, causing the waveform receiver to record the incorrect signal. If a wrong signal is captured, then the signal capture process for the current entry requires a reset. As a result, it takes around 50 min to collect 500 samples for each location. Second, the proposed system has been evaluated only under the LoS scenario within one environment. The proposed system’s performance may vary according to the landscape and structures in which it is installed.

## 6. Conclusions

In this research, we developed and tested a novel method that leveraged RSSI and AoA estimation to perform high-accuracy ranging tasks without high bandwidth with the help of ANN. The initial trial delivered a favorable outcome with the proposed system successfully reducing ranging errors under ten centimeters, which ranks at a high position compared to the systems used in the literature as we can see on Table 1. This indicates that high-precision indoor ranging without wide signal bandwidth or synchronization is possible. The advantage of this work over existing high-accuracy ranging systems (TOA/TDOA-based method) is that our proposed system is simple to deploy due to low complexity equipment and cost effective. While the proposed method does have some limitations, as discussed in Section 5, these issues can be resolved in future research.

## Figures and Tables

**Table 1:** Ranging accuracy in previous work.

Study	Method	Median Error	# of APs
Martin et al. [16]	RSSI-fingerprint	1.5 m	3
Achroufene et al. [17]	RSSI-ranging	1.13 m	5
Biehl et al. [18]	RSSI-fingerprint	0.94 m	7
Chen et al. [25]	AoA	1.5 m	1
Kumar et al. [26]	AoA	0.3 m	5
Xiong et al. [27]	AoA	0.23 m	6
Adege et al. [28]	ANN	0.5 m	7
Ibrahim et al. [29]	ANN	0.49 m	1
Adhikari et al. [30]	ANN	0.035 m	10

**Table 2:** Properties of ANN.

Properties	Value
Input	$P_{AoA}$, RSSI1, RSSI2, RSSI3
Output	# of Periods
Algorithm	Bayesian Regularization
Layers	30
Loss Function	mse

## Data Availability

Not applicable.

## Appendix A

**Table A1:** Lab Environment Test field coordinates (relative to AP).

Index	X	Y	AoA	Distance	Periods
1	0.49	1.77	15.62	2.16	39
2	0.99	1.82	28.78	2.36	43
3	1.67	1.74	43.6	2.64	48
4	2.3	1.74	52.89	3.08	56
5	2.8	1.71	58.23	3.44	63
6	0.45	2.36	10.79	2.68	49
7	0.96	2.33	22.13	2.78	51
8	1.56	2.31	34.02	3.01	55
9	2.1	2.33	42.02	3.32	61
10	2.75	2.38	49.22	3.78	69
